# Syphilis

**Published:** 1902-05

**Authors:** 


SYPHILIS. A Symposium of Special Contributions from Seventeen Different Writers. New York. E. B. Treat & Co. 1902. [Price, $1 00.]

The value of this small volume can scarcely be overestimated, and it is quite proper to say that no practitioner of medicine, general or special, should be without it. The names of the contributors are: L. Duncan Bulkley, Follen Cabot, Jr., Louis A. Duhring, Prof. Fournier, Eugene Fuller, E. B. Gleason, William S. Gottheil, Robert H. Greene, Norman B. Gwyn, Orville Horwitz, Edward L. Keyes, G. Frank Lydston, D. J. McCarthy, Thomas G. Morton, Boardman Reed, A. Robin and J. D. Thomas. It can be carried easily in the pocket and certainly is entitled to be called, *multum in parvo*. J. H. D.

